# Bioanalytical research and training in academia during the COVID-19 pandemic

**DOI:** 10.4155/bio-2020-0152

**Published:** 2020-06-26

**Authors:** Frank Klont, Gérard Hopfgartner

**Affiliations:** ^1^Life Sciences Mass Spectrometry, Inorganic & Analytical Chemistry, University of Geneva, Geneva, Switzerland

**Keywords:** academia, bioanalysis, COVID-19, education, research

The world is currently facing a crisis of unimaginable proportions due to the COVID-19 pandemic, and this novel coronavirus continues to pile misery on people of all ages, all across the world, and from all walks of life. In the past few months, governments have imposed unprecedented measures to halt the spread of the coronavirus, and these measures continue to impact daily lives and societies while also business operations and the economy in general. The field of bioanalysis is one of the many fields facing changes and challenges in the current crisis, which, fortunately, do not all strictly have a negative impact on laboratory operations, as was recently discussed by Pruim and Teekamp [1]. This article highlighted some of the impact on an industrial bioanalytical setting, and here we aim to complement their work by elaborating on the impact of the COVID-19 pandemic from the perspective of bioanalytical research and training in an academic laboratory.

## General measures

Many universities imposed their measures gradually, which frequently led to near-complete campus lockdowns. At first, steps were taken that dealt with the protection of people at increased risk for infection and with stimulating teleworking and distance learning. Soon after, however, teaching activities were moved completely online, and all research activities on university premises were suspended, with the exception of the maintenance of living organisms, COVID-19-related research and specific activities for which an abrupt suspension would lead to unacceptable and/or dangerous damages. Near-identical policies were thus pursued for teaching and research, which represent the key pillars of an academic institution. Consequently, ‘wet lab’ bioanalytical research came to a complete standstill at several universities, and the resulting circumstances thereby contrast with the situations in many industrial bioanalytical laboratories where laboratory activities could be continued, albeit in modified forms taking into account strict precautions.

## Impact on teaching staff

Probably the highest degree of adaptability, creativity and determination has been demanded from the teaching staff ever since the first pandemic-related measures were taken. Views on how teaching can and should be organized in these turbulent times keep changing, and universities are particularly struggling with the organization of exam sessions and practical training (e.g., practical classes, research internships and project internships). Our group, for example, was halfway through teaching an introductory practical class for first-year pharmacy students when on-site teaching was suspended. During this course, students were taught some analytical chemistry basics, which could be moved online with relative ease and without having to make too many compromises. This course was furthermore aimed at improving the students' laboratory skills, which can partly be addressed online via instructional videos and partly at later stages, for example, during follow-up practical classes in the next year. However, social distancing measures will likely remain in place for a while and will inevitably have an impact on how practical classes will be organized in the next year(s). Collectively, it is expected that teaching staff needs to continue acting upon the impact of the COVID-19 pandemic for several years, and their adaptability, creativity and tenacity will likely become even more determining for the quality of teaching at academic institutions.

## Impact on permanent staff

Permanent staff members, including research technicians, instrumentation engineers and electrical engineers, are the persons who keep research groups and projects going, and they are vital links in most, if not all bioanalytical laboratories. Their assistance may now be less required for resolving issues caused by research students who implemented experiments with any errors, yet they can play important roles in preparing for a restart of research activities when the coronavirus-related measures are eased. It may even be advisable to let them coordinate the entire restart process since PhD students and post-docs might permit themselves to perform a complete, unrestricted restart of research activities once they are allowed to gradually resume their experimental work. Ensuring safety of permanent staff must be high priority and will likely represent the beginning of restarting any activity in a bioanalytical research laboratory.

## Impact on PhD students & postdoctoral researchers

PhD students and postdocs typically work in a research laboratory for a limited number of years in which they aim to maximize their productivity in terms of gathering knowledge and expertise, publishing research findings and sometimes also securing research funding to allow them to conduct next-stage research projects. The temporary nature of their appointments typically comes along with considerable time pressure, which may intensify due to the COVID-19 pandemic. However, the impact of the current crisis on individual PhD students and post-docs in bioanalytical sciences largely depends on the stage and nature of their projects. For example, it is likely to be easier for first-year PhD students to pause their experiments and possibly start writing a review article, which may later serve as an introductory chapter in a PhD thesis, than it would be for finishing PhD students who needed the past few months to perform some final experiments to wrap up their PhD theses. Furthermore, it will be easier for researchers who spend most of their time analyzing data to continue their projects, than it will be for someone who normally spends the majority of the time within a laboratory. No one-size-fits-all strategies will therefore be adopted for dealing with individual situations during the lockdown and afterward, when activities on university premises may gradually be resumed. These situations need to be considered in light of the stage and nature of research projects while also taking into account the alternatives and opportunities that have become available in this period.

For example, a senior PhD student from our group was able to finalize all remaining data processing tasks via remote access software tools in the crisis period. In addition, she practiced and improved her PhD thesis presentation using video conferencing tools and she eventually defended her thesis in a setting where the PhD opposition committee members and the audience dialed in from remote locations via online video conferencing tools. Most of these tools were fortunately already in place within our department, yet the current situation did make it even clearer to us that we need to remain up-to-date with developments in information technology.

## Impact on bachelor's & master's students

Many universities pursued policies to move all on-site teaching activities to online alternatives for (at least) the remainder of the current academic year. These policies largely impacted the work of students conducting their bachelor's or master's research projects in an academic research group, while also of those carrying out research or project internships in nonacademic institutions. Admittedly, our field of research can offer alternative research programs that require limited to no physical presence on campus, including projects involving the processing of unpublished research data or data present in public research data repositories. Such projects may, however, contribute only marginally to expanding the students' knowledge and expertise on the practical aspects of bioanalysis, which frequently represent important attributes for next-stage positions in both industry and academia. The impact of moving to alternatives requiring little to no on-site physical presence may arguably be limited for master's students who could finish most of their practical work before the lockdown measures were imposed, to bachelor's students who will continue studying for a master's degree in the coming years, and to students working on bioinformatics-oriented projects. Still, large numbers of students are expected to face considerable challenges after their graduation as a result of the current pandemic, and these challenges may potentially have societal and economic consequences.

## Conclusion

We can only scratch the surface of the impact that the COVID-19 pandemic has on the practices of our academic bioanalytical laboratory, and we do not have a clear view on all challenges that lie ahead of us. Still, we can acknowledge that positive developments may come out of the current crisis and that the impact on our operations has been relatively small compared with, for example, the healthcare and hospitality industries. We should, however, not forget that academic research in many countries was among the first activities to be suspended and that experimental bioanalytical research often came to a complete standstill during the enforced lockdown measures. To this regard, we are confident that many lessons will be learnt from the current crisis and that universities, like many other institutions, will be able to keep a larger part of their operations going if/when a next crisis takes hold of our society in the future.

We are looking forward to resuming our experimental work, and we believe that imposed social distancing measures can readily be implemented within our department. We also believe that in these times, particular attention should be paid to mental health-related matters, notably because corresponding issues are (too) frequently observed among researchers in academia [2]. Particular attention should also be given to those who are finishing their university training during the current crisis. These talented academics will soon be entering the job market that has changed drastically during the COVID-19 pandemic, and we should do our utmost to prevent their skills from getting lost. Their talent may, for example, be utilized for realizing innovative analytical tools that are now particularly needed since countries need to ‘…test, test, test…’, as was recently advised by the director general of the World Health Organization, Dr Tedros Adhanom Ghebreyesus [3].
